# Varix Rupture Due to High-Flow Parasagittal Sinus Dural Arteriovenous Fistula: A Case Report and Literature Review

**DOI:** 10.7759/cureus.83760

**Published:** 2025-05-08

**Authors:** Koji Hirata, Kyoji Tsuda, Keishi Fujita, Eiichi Ishikawa, Yuji Matsumaru

**Affiliations:** 1 Department of Neurosurgery, Institute of Medicine, University of Tsukuba, Tsukuba, JPN; 2 Department of Neurosurgery, Ibaraki Seinan Medical Center Hospital, Sakai, JPN

**Keywords:** cortical venous reflux, dural arteriovenous fistula (davf), subcortical hemorrhage, superior sagittal sinus, varix

## Abstract

Parasagittal sinus dural arteriovenous fistulas (DAVFs) are rare and aggressive vascular malformations with a high risk of intracranial hemorrhage (ICH). We present the case of a 75-year-old man with altered consciousness, anisocoria, and left hemiparesis due to a large subcortical ICH. Imaging revealed a ruptured varix associated with a parasagittal sinus DAVF (Cognard type IV, Borden type III). The patient underwent emergency hematoma evacuation, followed by successful transarterial embolization via the contralateral middle meningeal artery (MMA) using N-butyl-2-cyanoacrylate. Despite complete angiographic obliteration, the clinical outcome was poor.　Parasagittal sinus DAVFs should be considered in the differential diagnosis of large subcortical hemorrhages. It should also be noted that certain cases may result in unfavorable clinical outcomes. When ipsilateral arterial access is unavailable, a contralateral approach via the MMA can be effective.

## Introduction

Dural arteriovenous fistula (DAVF) is a rare condition with an incidence of 0.16-0.51 per 100,000 adults per year [1-3]. However, due to recent advances in diagnostic technology, intracranial DAVFs have become more frequently diagnosed [4].

DAVFs involved with the superior sagittal sinus (SSS) have been reported in only up to 5% of DAVFs [5]. SSS-DAVFs were classified as sinus-type DAVF, which feature a fistulous point on the dura mater of the SSS, or non-sinus-type DAVF, which directly connect to the cortical vein. Non-sinus-type SSS-DAVFs are classified into convexity-DAVFs, falx-DAVFs, or parasagittal sinus DAVFs. Fistular flow in parasagittal sinus DAVFs drains directly into the cortical vein, leading to cortical venous reflux (CVR). These DAVFs are classified as Borden type III or Cognard type III or IV. Parasagittal sinus DAVFs have an aggressive clinical course because of CVR. Up to 42% of patients presented with intracranial hemorrhage (ICH) [6]. However, knowledge is limited on parasagittal sinus DAVFs because of few reviews and case series [5-7]. The large hematoma is clinically unusual but requires further case accumulation to determine its significance.

To our knowledge, no reports have associated substantial subcortical ICH with parasagittal sinus DAVF. Here, we report a case of parasagittal sinus DAVF that required transarterial embolization after hematoma removal and cranial decompression and review the relevant literature.

## Case presentation

**Figure 1:**
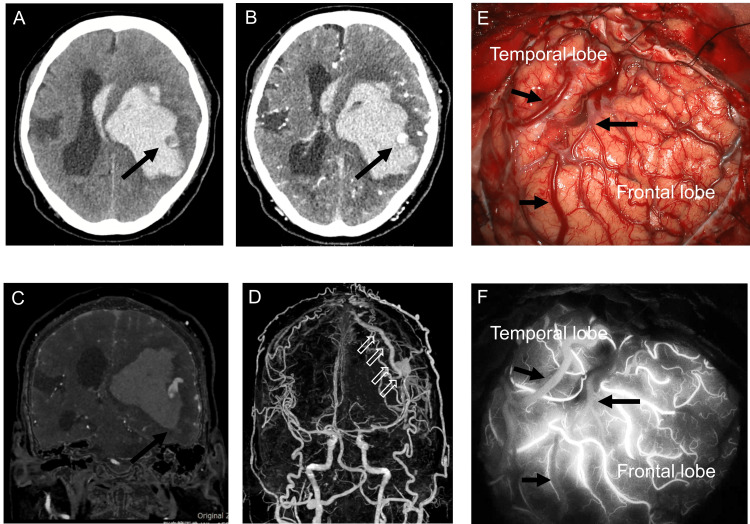
Noncontrast (a) and contrast (b, c) computed tomography revealing left subcortical intracerebral hemorrhage and varix (black arrow). Volume-rendering image (d) showing a dilated cortical vein (open arrow). (e) Intraoperative view during craniotomy. (f) Indocyanine green videoangiography findings. Black arrows indicate the dilated draining vein (“red vein”). The shunt point of the dural arteriovenous fistula (dAVF) was located outside the operative field, making simultaneous hematoma evacuation, decompressive craniotomy, and shunt dissection technically challenging

A 75-year-old man was admitted to the hospital with a consciousness disorder, anisocoria, and left hemiparesis. The patient had no history of head trauma, neurosurgical procedures, or hypertension, and no prior episodes of similar neurological symptoms. Laboratory evaluation revealed no evidence of coagulopathy. He was not taking any oral antithrombotic medications, and there was no relevant family history. His Glasgow Coma Scale score was 1-1-3. Computed tomography (CT) revealed a large left subcortical hemorrhage measuring 7.6 × 5.1 × 6.0 cm, accompanied by a midline shift (Figure 1a).

**Figure 2:**
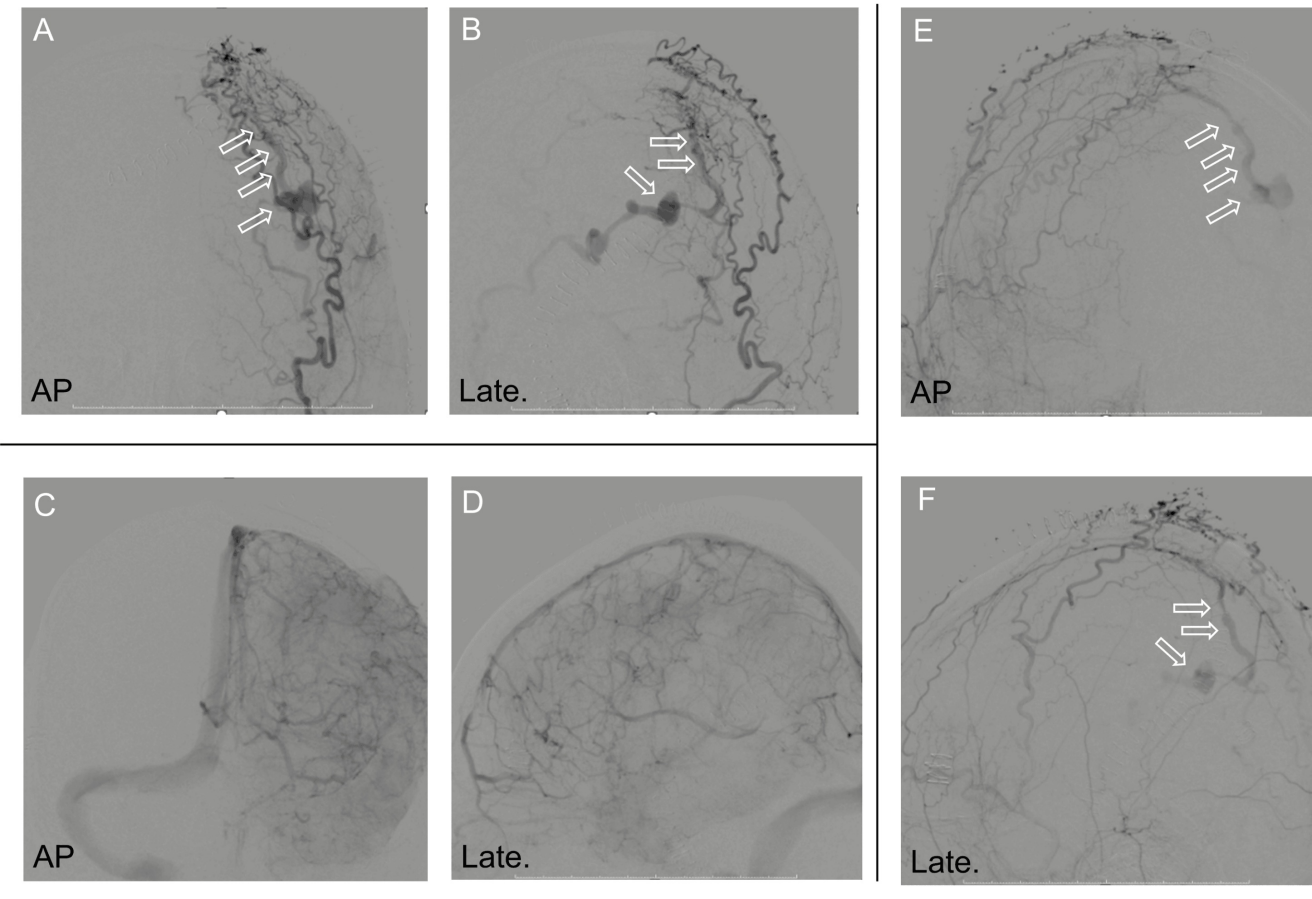
Anteroposterior (a) and lateral (b) views of the left external carotid angiogram showing a parasagittal sinus dural arteriovenous fistula and dilated cortical vein (open arrow). Anteroposterior (a) and lateral (b) views of the left internal carotid angiogram showing no arteriovenous shunt or pseudophlevitic pattern and the patency of the superior sagittal sinus. Anteroposterior (e) and lateral (f) views of the right external carotid angiogram showing the same parasagittal sinus dural arteriovenous fistula and dilated cortical vein (open arrow)

Three-dimensional (3D) CT angiography showed a varix of the cortical veins, which was the cause of the bleeding (Figures 1b-1d). Partial hematoma removal was performed due to incomplete hemostasis of the varix, along with cranial decompression. Due to the shunt point being situated in the parietal paracentral region, concurrent surgical dissection of the shunt was deemed technically challenging during the emergency procedures for intracerebral hematoma evacuation and decompressive craniotomy. Although postoperative CT showed rebleeding, because intracranial pressure and anisocoria improved, conservative treatment was performed. Finally, digital subtraction angiography (DSA) revealed parasagittal sinus DAVFs with direct cortical venous drainage with venous ectasia. The feeders were the bilateral occipital artery, contralateral superior temporal artery (STA), and middle meningeal artery (MMA). However, the ipsilateral STA and MMA did not feed into the DAVFs because these had already been cut during the surgery. The DAVFs had a single drainer at the cortical vein. The SSS was patent and did not communicate with the DAVFs. No pseudophlebitic pattern was observed on the medullary vein (Figure 2).

**Figure 3:**
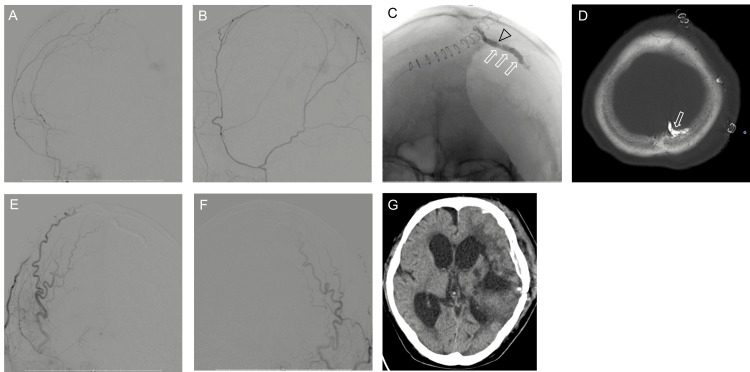
Anteroposterior (a) and lateral (b) views of the right middle meningeal artery angiogram showing the access route to the shunt pouch. Craniography (c) and computed tomography (d) showing the tip of the microcatheter in the venous pouch (open arrowhead) and the cast of N-butyl-2-cyanoacrylate (open arrow). Anteroposterior view of the right (e) and left (f) external carotid artery angiograms after treatment demonstrating no arteriovenous shunt. Follow-up computed tomography (g) showing no rebleeding

The diagnosis was parasagittal sinus DAVFs Cognard type IV and Borden type III. The patient underwent transarterial embolization (TAE) via the contralateral MMA because the right MMA had been transected during the craniotomy. An 8-Fr ASAHI FUBUKI guiding catheter (Asahi Intecc, Nagoya, Japan) was inserted into the right external carotid artery. Then, a 3.4-Fr TACTICS distal access catheter (Technocrat Corporation, Aichi, Japan) was navigated into the right MMA. A DeFrictor Nano Catheter (Medico’s Hirata) with ASAHI CHIKAIX10 (Tokai Medical Products, Aichi, Japan) was advanced via the anterior convexity branch of the MMA on the right side, across the midline, and toward the venous pouch located laterally (Figures 3a, 3b).

Microangiography showed the microcatheter in the venous pouch. TAE with 17% N-butyl-2-cyanoacrylate (NBCA) was performed (Figure 3c). Left/right common carotid angiography showed that the DAVFs had disappeared, while CT revealed the NBCA was in the venous pouch (Figure 3d). Residual intracerebral hematoma was removed endoscopically. On follow-up, DSA showed complete occlusion of the DAVFs, while CT showed no rebleeding (Figures 3e-3g). The patient underwent rehabilitation, but the level of consciousness did not improve, and the modified Rankin Scale (mRS) score was 5 at the time of discharge.

## Discussion

The differential diagnosis of extensive cerebral subcortical hemorrhage should include parasagittal sinus DAVF. The case of parasagittal sinus DAVF with a poor prognosis must be noted and considered. When the ipsilateral approach for TAE is complex, the contralateral approach through the MMA might provide patent access to the fistulous point.

Varix rupture associated with DAVF Borden type III causes massive parenchymal hemorrhage with cerebral herniation. Arteriovenous shunting through the fistula induces an arterialized flow into the normally low-pressure venous system, causing ICH. Venous hypertension created a venous varix. The cause of bleeding was generally venous congestion and a ruptured medullary vein, whereas some cases reported varix rupture [8]. Patients may suffer from an abrupt onset of subarachnoid hemorrhage when the varix ruptures. The medullary vein did not cause subcortical ICH because no venous congestion or pseudophlebitic pattern on the medullary vein occurred. A literature review on parasagittal sinus DAVFs revealed that 42% of cases presented with ICH [6]. A retrospective study of 15 patients with parasagittal DAVFs reported that five (26%) of 19 patients had ICH [5]. However, an eight-case series included no patients with ICH [7]. The frequency of ICH varied, and several symptoms were associated with parasagittal sinus DAVF, including hemiparesis, headache, seizures, visual symptoms, dementia, aphagia, and tinnitus. The parasagittal sinus dAVF had an aggressive clinical course because of direct CVR, which was Cognard III or IV. Although ICH occurred in 14 parasagittal sinus DAVFs, the bleeding volume was insignificant, with nine intraparenchymal hemorrhages, two subarachnoid hemorrhages, and one subdural hemorrhage (two unavailable; Table 1) [5,6].

**Table 1:** Characteristics of patients with parasagittal sinus dural arteriovenous fistula

Characteristics of patients with parasagittal sinus DAVF									
Author	year	N	Age/sex	Symptoms	Cognard	Hemorrhage	Size, cm	Cerebral herniation	Clinical outcome
Halbach et al. [9]	1988	1	N/A	Aphasia	IV	IPH	N/A	No	Full rec.
Barnwell et al. [10]	1991	1	48/F	N/A	N/A	SDH	N/A	NA	Full rec.
Kurl et al. [11]	1996	1	46/M	Headache	IV	IPH	N/A	No	Full rec.
Hurst [12]	1998	1	38/M	Headache	IV	IPH	5x2	No	N/A
Hurst et al. [12]	1998	1	19/M	Headache	N/A	SAH	N/A	No	N/A
Hurst et al. [12]	1998	1	62/M	Headache	IV	IPH	5 x 2	No	N/A
Bavinzski et al. [13]	1999	1	57/M	Hemiparesis	III	IPH	N/A	No	Full rec.
Chai and Wang [14]	2011	1	37/M	N/A	IV	IPH	5x2	No	Full rec.
Gross and Du [15]	2013	1	52/F	N/A	N/A	NA	N/A	No	Full rec. mRS0
Gross and Du [15]	2013	1	52/M	N/A	N/A	NA	N/A	No	Rec partially mRS4
Song et al. [16]	2017	1	63/F	Headache	IV	SAH and IVH	N/A	No	Full rec.
Kotsugi et al. [17]	2019	1	64/M	Hemiparesis	III	IPH	N/A	No	Full rec.
Chavan et al. [6]	2021	1	40/M	Headache	IV	IPH, IVH	3.8 x 2.6 x 3.5	No	Full rec.
Alexander et al. [7]	2024	8	34-79	Vertigo, ataxia, paresis, dysarthria, pulsatile bruit, brain fog, aphasia	III: 7 IV: 1	ICH: 0	N/A	No	rec. 8/8 (100%)
Kee et al. [5]	2024	15	N/A	N/A	IV	ICH	N/A	No	rec. 15/15 (100%)
Present case	2024	1	75/M	Unconsciousness hemiparesis anisocoria	IV	ICH	7.6 x 5.1 x 6.0	Yes	mRS5
NA: not available; IPH: intraparenchymal hemorrhage; SDH: subdural hemorrhage; SAH: subarachnoid hemorrhage; IVH: intraventricular hemorrhage; mRS: modified rankin scale; rec.: recovery									

In the present case, the patient presented with coma and anisocoria in the emergency room. Head CT revealed a sizable subcortical ICH and cerebral herniation. Although parasagittal sinus DAVFs with a large hematoma have not been reported, the differential diagnosis of extensive cerebral subcortical hemorrhage associated with cerebral herniation includes parasagittal sinus DAVF.

The present case underwent emergency hematoma removal, but rebleeding occurred soon after surgery. The rate of rebleeding of Borden type III DAVF is high [18]. Although no data are available on the rate of rebleeding after hematoma removal in Borden type III DAVF-associated bleeding, rebleeding after hematoma removal should be strictly considered, and careful postoperative management must be applied.

The clinical course of patients with parasagittal sinus dAVF is good: 24/25 (96%) with full recoveries and one at mRS 4 (three unavailable) [5-7]. The clinical course of the present case was poor, mRS 5 at discharge, despite undergoing emergency hematoma removal and cranial decompression. A previous report found no permanent complications resulting from treatment, and presenting symptoms resolved in symptomatic patients [7]. Given the possibility of severe disability, a treatment strategy must be prepared as soon as possible for parasagittal sinus DAVF. Prompt therapeutic intervention is warranted for Borden type III DAVF due to their aggressive clinical behavior. Informed consent is necessary for severe progression.

A transarterial approach was performed for parasagittal sinus DAVF because of the obliterated venous route to the shunted pouch. A previous report described TAE using liquid embolic material via the ipsilateral MMA, the primary treatment approach for parasagittal sinus DAVF. Complete angiographic occlusion was achieved in 93.33% of cases [5]. The arterial access route for embolization was decided based on vessel tortuosity and the size of the involved vessels. Because the transosseous route to the shunted pouch was difficult to access, a dural arterial feeder with a straighter course was first selected for embolization because of the ease of catheter navigation. However, because the ipsilateral MMA was transected during the craniotomy, we used the contralateral MMA for the approach route. The contralateral approach to the fistulous point of the parasagittal sinus DAVF was challenging. Furthermore, microsurgical disconnection is effective for parasagittal sinus DAVF with Cognard III or IV. However, we performed endovascular therapy because it was less invasive than direct surgery. Surgeons may consider the contralateral approach route in treating parasagittal sinus DAVF before disconnecting the draining vein during direct surgery. The microcatheter can reach the closure of the shunted pouch as the device advances, which enables safe and reliable embolization. A case series found that the fistulous point of the parasagittal sinus DAVF was near the junctional zone between the cortical vein and the SSS [5,7]. In the present case, the fistulous point was on the dura mater at convexity because the microcatheter advanced to the contralateral convexity, and its tip entered the shunted pouch. Thus, the fistulous point was the same as those in previous reports [5,7].

## Conclusions

Parasagittal sinus DAVFs are rare but aggressive vascular lesions with a high risk of ICH, particularly when associated with cortical venous reflux and venous ectasia. This case highlights the potential for massive subcortical hemorrhage due to varix rupture in parasagittal sinus DAVF, a presentation that has rarely been documented in the literature. Clinicians should maintain a high index of suspicion for DAVF in cases of unexplained extensive subcortical hemorrhages, particularly when conventional risk factors such as hypertension or trauma are absent.

Timely diagnosis using CT angiography and DSA is crucial, as is selecting an appropriate treatment strategy. Although TAE via the ipsilateral MMA is a standard approach, a contralateral MMA route may be a viable alternative in cases where the ipsilateral pathway is inaccessible. Our experience suggests this technique can achieve complete fistula obliteration even under anatomically challenging conditions.

Nevertheless, despite successful radiographic outcomes, clinical recovery may be limited, particularly in cases presenting with coma or herniation. Early identification and intervention, as well as careful intraoperative planning to preserve potential access routes, are key to improving patient outcomes. This case emphasizes the importance of rapid multidisciplinary management and contributes to the growing body of evidence regarding the diverse presentation and management strategies for parasagittal sinus DAVFs.
